# Simultaneous detection of sepsis host response biomarkers in whole blood using electrochemical biosensor

**DOI:** 10.1002/btm2.10310

**Published:** 2022-03-17

**Authors:** Ambalika S. Tanak, Abha Sardesai, Sriram Muthukumar, Shalini Prasad

**Affiliations:** ^1^ Department of Bioengineering University of Texas at Dallas Dallas Texas USA; ^2^ Department of Computer engineering University of Texas at Dallas Dallas Texas USA; ^3^ EnLiSense LLC Allen Texas USA

**Keywords:** electrochemical sensing, host response biomarkers, multiplexed detection, point‐of‐care biosensor, sepsis detection, whole blood testing

## Abstract

Sepsis is a silent killer, caused by a syndromic reaction of the body's immune system to an infection that is typically the ultimate pathway to mortality due to numerous infectious diseases, including COVID‐19 across the world. In the United States alone, sepsis claims 220,000 lives, with a dangerously high fatality rate between 25% and 50%. Early detection and treatment can avert 80% of sepsis mortality which is currently unavailable in most healthcare institutions. The novelty in this work is the ability to simultaneously detect eight (IL‐6, IL‐8, IL‐10, IP‐10, TRAIL, d‐dimer, CRP, and G‐CSF) heterogeneous immune response biomarkers directly in whole blood without the need for dilution or sample processing. The DETecT sepsis (Direct Electrochemical Technique Targeting Sepsis) 2.0 sensor device leverages electrochemical impedance spectroscopy as a technique to detect subtle binding interactions at the metal/semi‐conductor sensor interface and reports results within 5 min using only two drops (~100 μl) of blood. The device positively (*r* >0.87) correlated with lab reference standard LUMINEX for clinical translation using 40 patient samples. The developed device showed diagnostic accuracy greater than 80% (AUC >0.8) establishing excellent specific and sensitive response. Portable handheld user‐friendly feature coupled with precise quantification of immune biomarkers makes the device amenable in a versatile setting providing insights on patient's immune response. This work highlights an innovative solution of enhancing sepsis care and management in the absence of a decision support device in the continuum of sepsis care.

## 1 INTRODUCTION

Sepsis and sepsis‐related complications like multi‐organ failure is indeed a substantial challenge on the healthcare systems and a major research concern for scientist around the world. Sepsis pathogenesis remains unclear, despite significant fundamental research, and clinical trials.^1^ Sepsis is increasingly being recognized as an incredibly diverse disease resulting from abnormalities within the inflammatory pathways. In the intensive care unit (ICU), sepsis is one of the most prevalent causes of mortality and in the United States alone, sepsis is responsible for up to half of all in‐hospital fatalities. The global incidence of hospital‐related sepsis in adults is estimated to be over 270 per 100,000, with an astonishing 26% overall death rate. There are 19.4 million cases and 5.3 million fatalities worldwide each year, excluding sepsis incidence amongst children and that which occur outside the hospital.^2^ In the presence of infection through pathogen, sepsis is developed as a systemic complication of the host immune system.^3^ The pathogenic invader is the catalyst, while the overzealous immune response of the host is to blame for the extensive organ damage that is a hallmark of the disease. The identification of several key biomarkers that enable improved risk stratification and treatment decision‐making has stemmed from a better understanding of the inflammatory events that contribute to host tissue damage in sepsis. There is substantial evidence that monitoring key biomarkers in the hyperinflammatory cytokine storm and acute phase response provides valuable diagnostic and prognostic indicators of disease progression. Interestingly, critically ill COVID‐19 patients are known to have sepsis, often accompanied by infection and organ failure with elevated concentrations of cytokines ultimately leading to tissue damage, need for a mechanical ventilator, and eventually death.^4^ Cytokines are substances released by innate and adaptive immune system components that serve as signaling pathways or activators of the inflammatory response, which play a pivotal role in the development of sepsis.^5^

Comprehensive mapping of the biomolecular milieu at a particular time point, is required for the development of viable treatment approaches. Previous research has identified a connection between blood levels of various cytokines, the severity of inflammatory response, and sepsis prognosis.^6^, ^7^, ^8^, ^9^ The present workflow in a clinical setting follows a systemic process depending on the technology and manpower available to diagnose patients with sepsis. If a patient remotely exhibits early indications of sepsis (i.e., SIRS criteria) antibiotics are immediately administered to target a variety of infectious sources before a disease is diagnosed.^10^, ^11^, ^12^ Although this may enhance patient survival, such antibiotic regimens are futile and lead to antibiotic resistance.^13^, ^14^ Parallelly, a large volume of blood samples is collected to identify the causative pathogens and fine‐tune the antibiotic prescription for the patient. A bacterial culture, gram staining, and drug resistance test are amongst the few clinical tests used to verify whether the treatment successfully limits pathogen proliferation. The major disadvantage with these methods is that results arrive days after the patient is hospitalized, which may lead to missing out on a valuable timeframe for accurate medical diagnosis and planning effective interventions. The remaining techniques for quantifying sepsis‐related biomarkers, such as flow cytometry and lactate tests, have a tendency of providing an inadequate diagnosis in most scenarios because they often require large sample volume, have constrained detection ranges, are difficult to discern results, which only delays prognosis from trained clinicians, limiting their usage in resource‐constrained environments across the world um.^15^, ^16^, ^17^, ^18^ Although some of the commercial point‐of‐care sepsis technologies have managed to improve their reliability and efficiency in clinical tests, none of them have multiplexing capabilities any further than their focused primary biomarkers class, limiting their ability to obtain an extensive sepsis immunological patient profile.

A solution to this everlasting problem is the development of a point‐of‐care biosensing device with the capability to overcome all the underlying issues with the existing testing methodologies. To this direction, we have developed a multiplexed panel of simultaneously detecting eight crucial biomarkers by using merely two drops (~100 μl) of undiluted whole blood to rapidly assess the patient immune response and project the possibility of a patient undergoing sepsis, rapidly with a sample to result turnaround time of 5 min. The effort to bring results closer to the patient bedside is propelled by designing miniaturized portable hardware coupled with sensitive electrochemical impedance spectroscopy (EIS) techniques to assist clinicians to monitor disease progression and provide guided treatment. Our vision is to develop a sensing device that can appease the ASSURED (Affordable, Sensitive, Specific, user‐friendly, Rapid and Robust, Equipment free, and Deliverable to end‐users) criteria set out by the World Health Organization which could also be useful anywhere.^19^, ^20^ The developed device is specially designed to be effective in resource‐replete conditions where the objective is to attain quick test results, with minimal sample handling, along with a projection of disease severity with the help of a machine learning model to aid in the planning of an emergency intervention.

## 2 RESULTS

### 2.1 Spike and recovery

**FIGURE 1:**
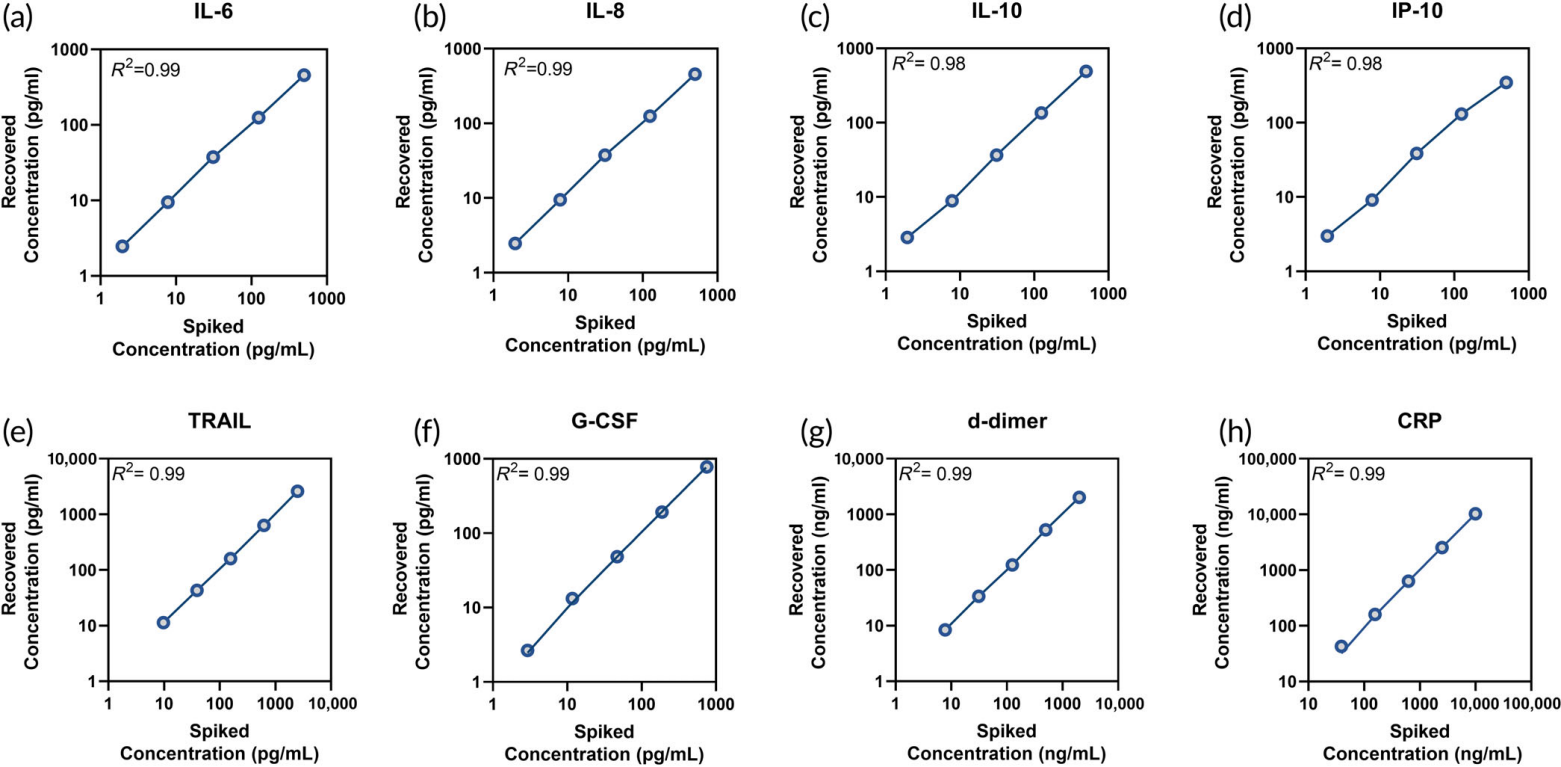
(a–h). Spike and recovery in whole blood sample using *n* = 3 sensors to demonstrate assay linearity using DETecT Sepsis 2.0 device for IL‐6, IL‐8, IL‐10, IP‐10, TRAIL, G‐CSF, d‐dimer, and CRP with *R* ^2^ >0.98

The blood panel with eight biomarkers each has individual standard curves containing a wide dynamic range to cover healthy and sick individuals with varying dose concentrations. The efficacy of the direct electrochemical technique targeting (DETecT) Sepsis 2.0 device was done by spiking a known concentration to the blood matrix and measuring the concentration using the calibrated curve for each biomarker (Figure 1a–h). The recovery of the spiked sample was determined by comparing it to calibrated dose response curve for individual biomarkers. Mean recovery concentration for the blood panel biomarkers was 105 ± 6% which lies within the accepted assay range according to the CLSI standards.^21^ When compared individually, the coefficient of determination, *R* ^2^ >0.97 implies the assay for the sensor device is linear with negligible matrix effect.

### 2.2 Specificity

**FIGURE 2:**
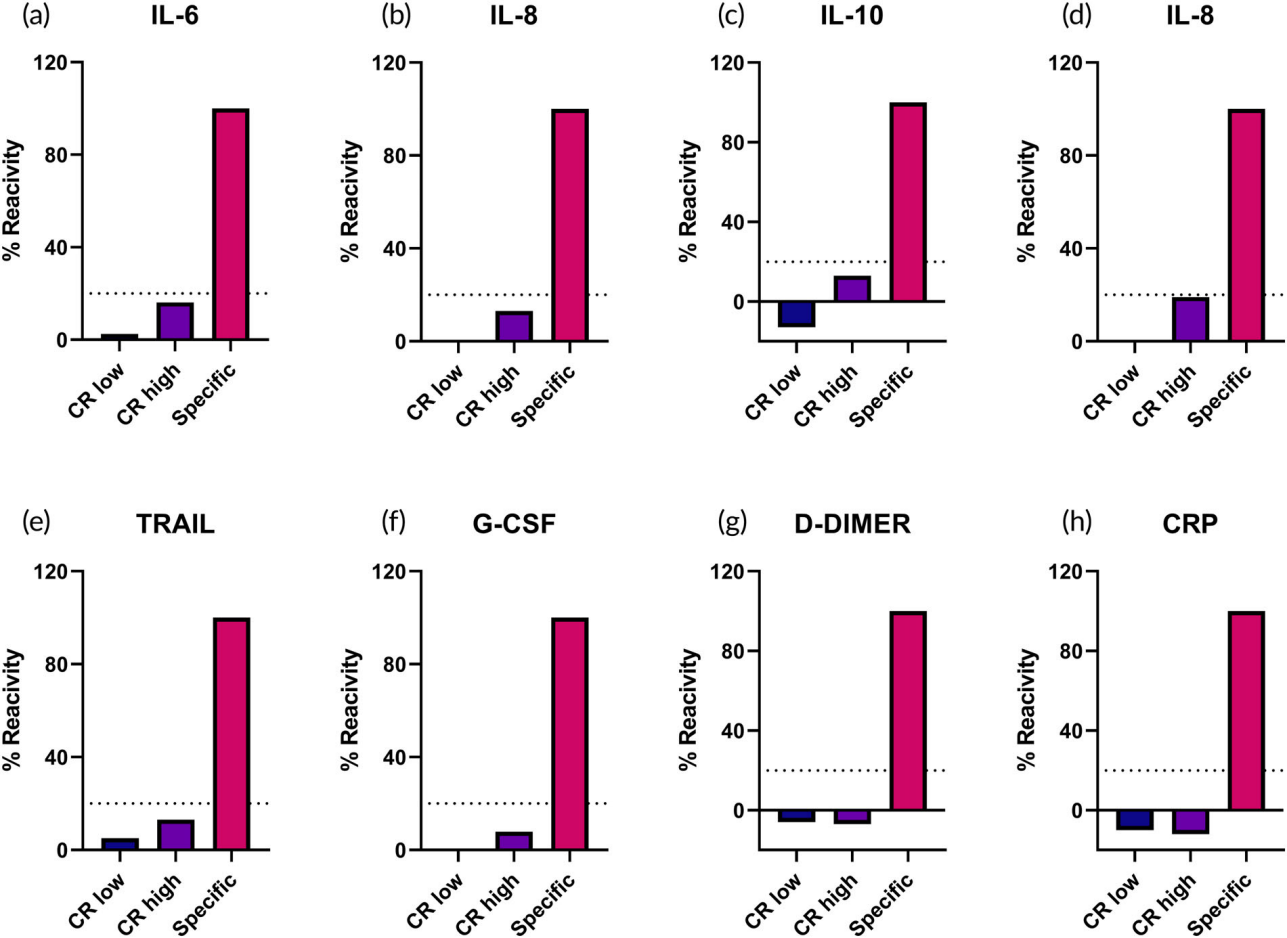
(a–h). The specificity of DETecT Sepsis 2.0 device for each of the target biomarker was evaluated using cross reactivity study.CR high: high concentration of BSA. CR low: low concentration of BSA. All the analytes are spiked in whole blood buffer matrix

Analytical specificity and selectivity of the DETecT Sepsis 2.0 device was evaluated by cross‐reactivity study using bovine serum albumin (BSA) in whole blood buffer matrix. Every analyte was tested with low concentration of BSA followed by high concentration of BSA depending on the target analyte's dynamic range. This was followed by adding low concentration of target‐specific analyte and the signal response was measured. Percent reactivity was calculated depending on the signal response for individual target analytes. As seen in Figure 2a–h, DETecT Sepsis 2.0 device was able to distinguish the specific target signal with ~100% reactivity and the nonspecific signal accounted for less than ~10% reactivity. When evaluating actual clinical samples, where the concentration of the actual analyte might be significantly lower than those of the nonspecific molecule, the biosensor's selectivity is essential. Overall reactivity less than 10% for the nonspecific BSA molecule indicates that DETecT Sepsis 2.0 device selectively binds to the target analyte with minimal interference from the nonspecific molecules present in whole blood samples.

### 2.3 Clinical validation of DETecT Sepsis 2.0 device with reference standard LUMINEX using whole blood patient samples

**FIGURE 3:**
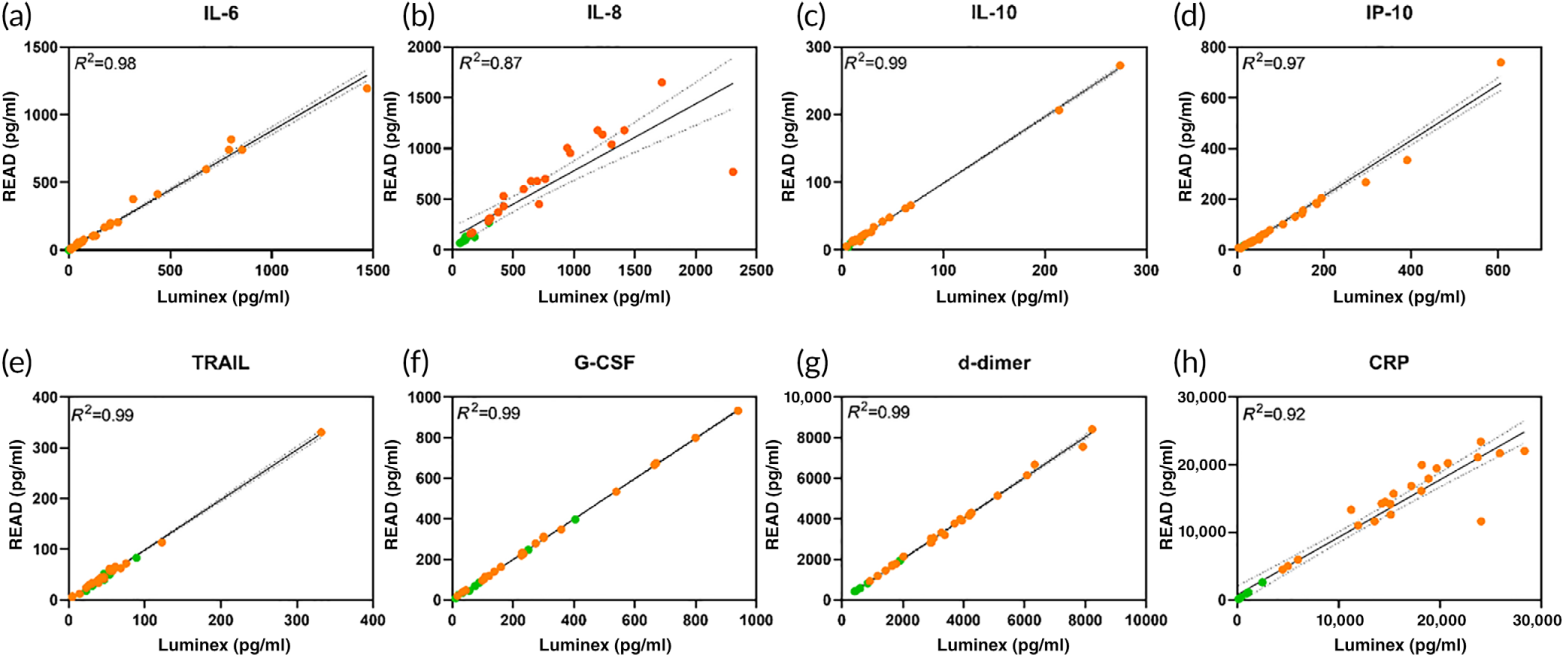
(a–h). Clinical validation of blood panel using DETecT Sepsis 2.0 device. The DETecT Sepsis 2.0 device was validated using standard LUMINEX as a reference method against *n* = 40 patient samples in the whole blood matrix. All biomarkers showed positive correlation with a coefficient of determination (*R* ^2^ >0.87). Green indicates healthy controls and orange represents septic samples

It is crucial to understand and validate the performance of the developed sensor platform with an existing reference method and measure the accuracy of the sensor. For clinical validation, 40 patient blood samples were measured using the DETecT Sepsis 2.0 device and reference LUMINEX standard method (Figure 3a–h).

**FIGURE 4:**
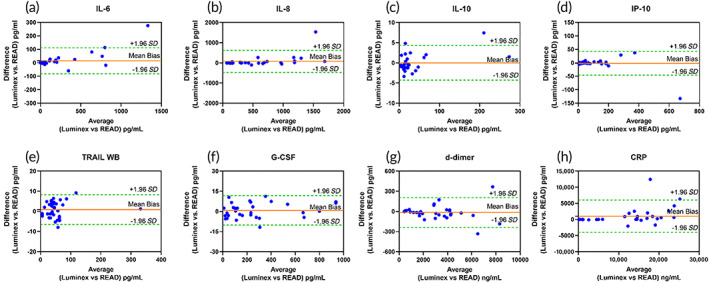
(a–h). Bland–Altman analysis comparing the developed device and reference Luminex standard using *n* = 40 patient blood samples

The concentrations measured by both methods correlated positively with a coefficient of determination (*R* ^2^ >0.97) for IL‐6, IL‐10, IP‐10, TRAIL, G‐CSF, and d‐dimer. Whereas IL‐8 and CRP correlated well with an *R* ^2^ >0.87. Additionally, the wide dynamic range ensures to capture both the healthy and the diseased state of the patient which can be useful as a monitoring device. To evaluate the preclinical utility of the developed sensor platform, it is essential for the sensors performance to agree with current lab standards. Hence, we compared the performance of DETecT Sepsis 2.0 device with LUMINEX using Bland–Altman analysis as seen in Figure 4a–h. The difference between each pair is plotted on the y axis while the average of each pair of measurements is plotted on the x axis. Low mean bias between the two methods shows a good degree of agreement between Luminex reference standard and DETecT Sepsis 2.0 device. Individual mean bias values can be found in Table S1. Since all the points except a few lie well within the limit of agreement (±1.96 *SD*), the two methods are in agreement. The dispersion of the points is minimally observed, and the points are reasonably near the mean bias line. Mean bias and the limit of the agreement provide information on the usability of the new measuring method as a quantifiable measure. The data measured across the blood panel biomarkers show equal distribution across the mean bias in both positive and negative directions. This indicates not one method overpredicts or underpredicts the concentration values.

**FIGURE 5:**
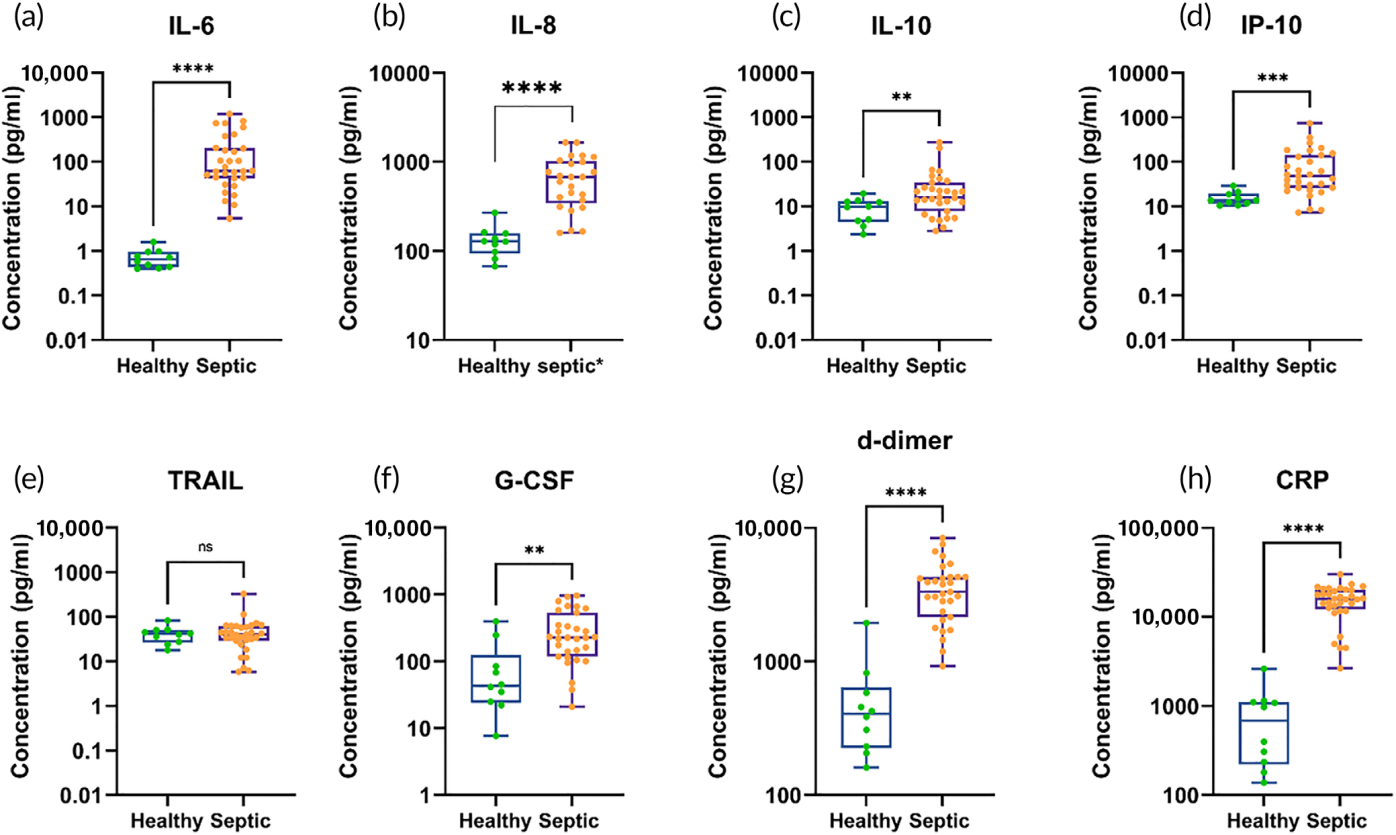
(a–h) Classification of infectious cytokine panel using *n* = 40 patient blood samples into healthy and septic patient cohort for IL‐6, IL‐8, IL‐10, IP‐10, TRAIL, G‐CSF, d‐dimer, and CRP. Statistical analysis was performed using Mann–Whitney *U* test to determine the significance between healthy and septic patient cohort. ns, no significance, **p <0.01, ***p <0.001, ****p <0.0001

Sepsis is a complicated disease with dysregulated immune response, measuring it with a single biomarker may not be informative. To know how sepsis affects the patient's immune response, it is necessary to look at a different group of biomarkers that can provide valuable insights. Therefore, we focused on cytokines, chemokines, and infectious biomarkers simultaneously to gauge the active patient state. Quantification of IL‐6, IL‐8, IL‐10, IP‐10, TRAIL, G‐CSF, d‐dimer, and CRP was done using DETecT Sepsis 2.0 device with 10 healthy whole blood control samples and 30 septic whole blood patient samples (Figure 5a–h). One hundred microliter of patient whole blood samples was directly added to the DETecT Sepsis 2.0 device without dilution or additional sample preparation. The mean blood concentration for IL‐6 was 0.7 ± 0.37 pg/ml and that of the septic cohort was 214 ± 297 pg/ml. IL‐8 showed a mean concentration of 135 ± 55 pg/ml for healthy and 706 ± 401 pg/ml for septic patients. Healthy whole blood concentrations for IL‐10 and IP‐10 were 9 ± 5.37 pg/ml and 15 ± 5.8 pg/ml, whereas septic IL‐10 and IP‐10 blood concentration were 34 ± 54 pg/ml 102 ± 144 pg/ml, respectively. Similarly, G‐CSF mean healthy concentration was 97 ± 125 pg/ml and patients with sepsis had a blood concentration of 320 ± 264 pg/ml. D‐dimer and CRP significantly classified healthy from the septic cohort with mean healthy blood concentration of 551 ± 556 pg/ml and 818 ± 758 pg/ml while septic blood concentration from 3636 ± 1818 ng/ml and 15,481 ± 6122 ng/ml, respectively, with no overlapping interquartile ranges. The multiplexed panel of eight biomarkers showed statistical significance between healthy controls against the septic patient cohort, with TRAIL as an exception. TRAIL has a negative trend where the healthy cohort shows a higher mean concentration of 42 pg/ml and the septic cohort shows a slightly lower mean concentration of 37 pg/ml. TRAIL is a potent inducer of cell death. Lower levels of TRAIL have been associated with an increased possibility of organ dysfunction, septic shock, and a higher rate of in‐hospital mortality.^22^, ^23^ Although plasma levels of TRAIL septic samples showed the capability to distinguish healthy from the diseased patient group,^24^ there are many factors associated with poor significance in whole blood samples.

### 2.4 Diagnostic Accuracy of DETecT Sepsis 2.0 device

**FIGURE 6:**
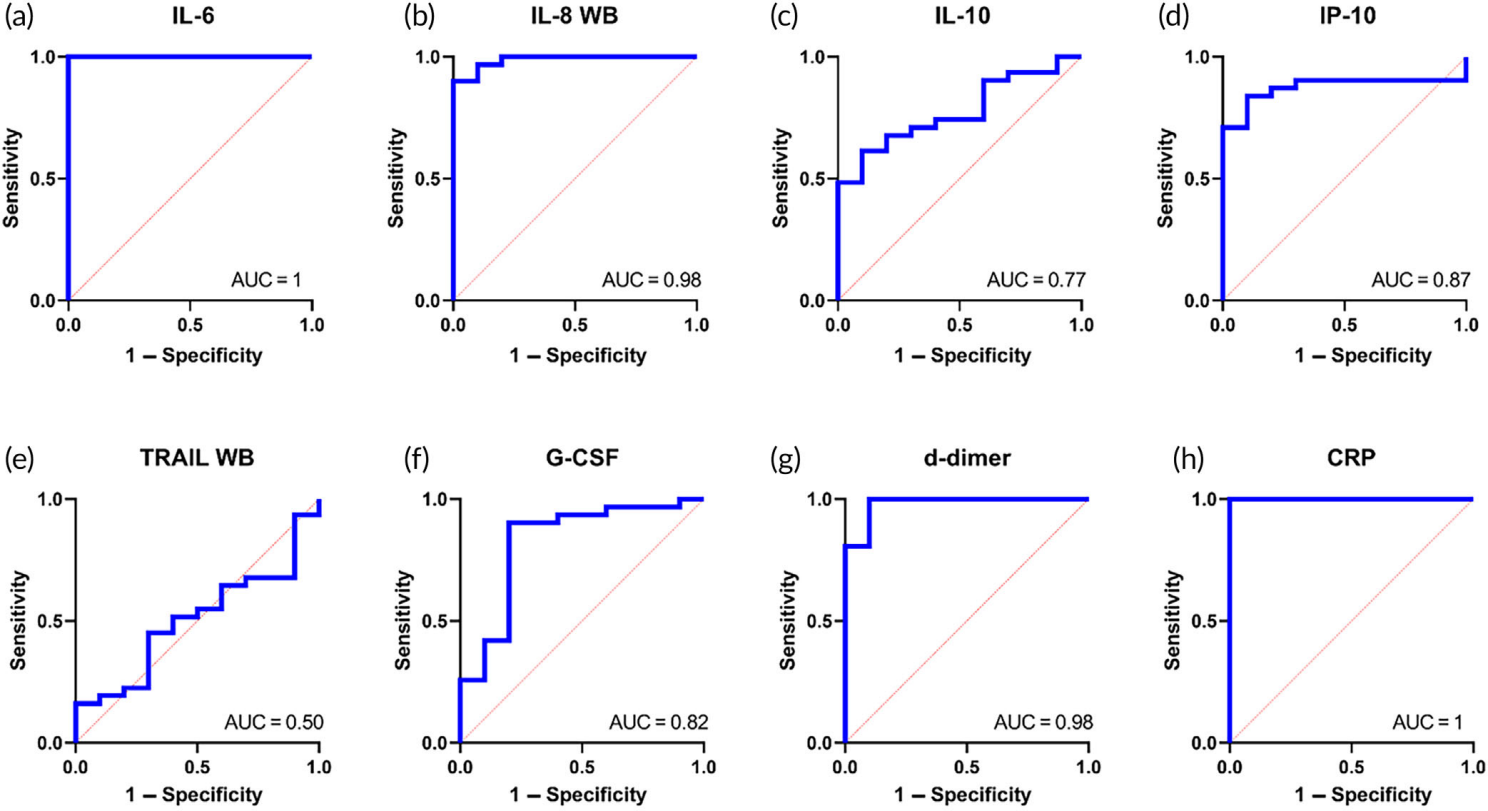
(a–h) Receiver operating characteristic curve for blood panel biomarkers using DETecT Sepsis 2.0 device. Areas under the curve for the individual biomarker is labeled at the bottom right of each graph

The utility of DETecT Sepsis 2.0 device in a clinical setting was tested using a receiver operating curve. It is essential for the device to identify the specificity and sensitivity of the device to minimize false‐positive results. The area under the curve (AUC) helps to evaluate the diagnostic performance of the test device. The ability of the device to identify patients with sepsis positively is termed device sensitivity. On the contrary, in AUC, specificity is defined as the device's capability to identify nonseptic patients as negative. As seen in Figure 6, IL‐6 and CRP had the highest discriminative value with an AUC of 1 (95% Confidence interval of 1–1) followed by IL‐8 and d‐dimer with an AUC of 0.98 (CI 0.93–1). IL‐6 had a sensitivity of 100%, and a specificity of 80% with a cut‐off 0.97 pg/ml was used to differentiate healthy from the septic patient cohort. CRP at a cut‐off value of 1882 ng/ml had a sensitivity of 100% and specificity of 90%. For IL‐8 had a sensitivity of 96% at a cut‐off value set as 161.6 pg/ml and specificity of 80% to distinguish healthy from the septic cohort. With a cut‐off at 872 ng/ml, d‐dimer showed 96% sensitivity and 90% specificity. IP‐10 and G‐CSF showed moderate discriminative value with an AUC of 0.87 (CI: 0.76–0.98) and 0.82 (CI:0.65–0.99), respectively. IP‐10 had 90% sensitivity and 70% specificity at a cut‐off at 16.26 pg/ml followed by 93% sensitivity and 60% specificity at a cut‐off value of 46.43 pg/ml for G‐CSF. Overall, the AUC for the blood panel biomarkers was above 0.90 for IL‐6, IL‐8, d‐dimer, and CRP which can be used as reliable biomarkers o differentiate healthy from the septic patient cohort.

**FIGURE 7:**
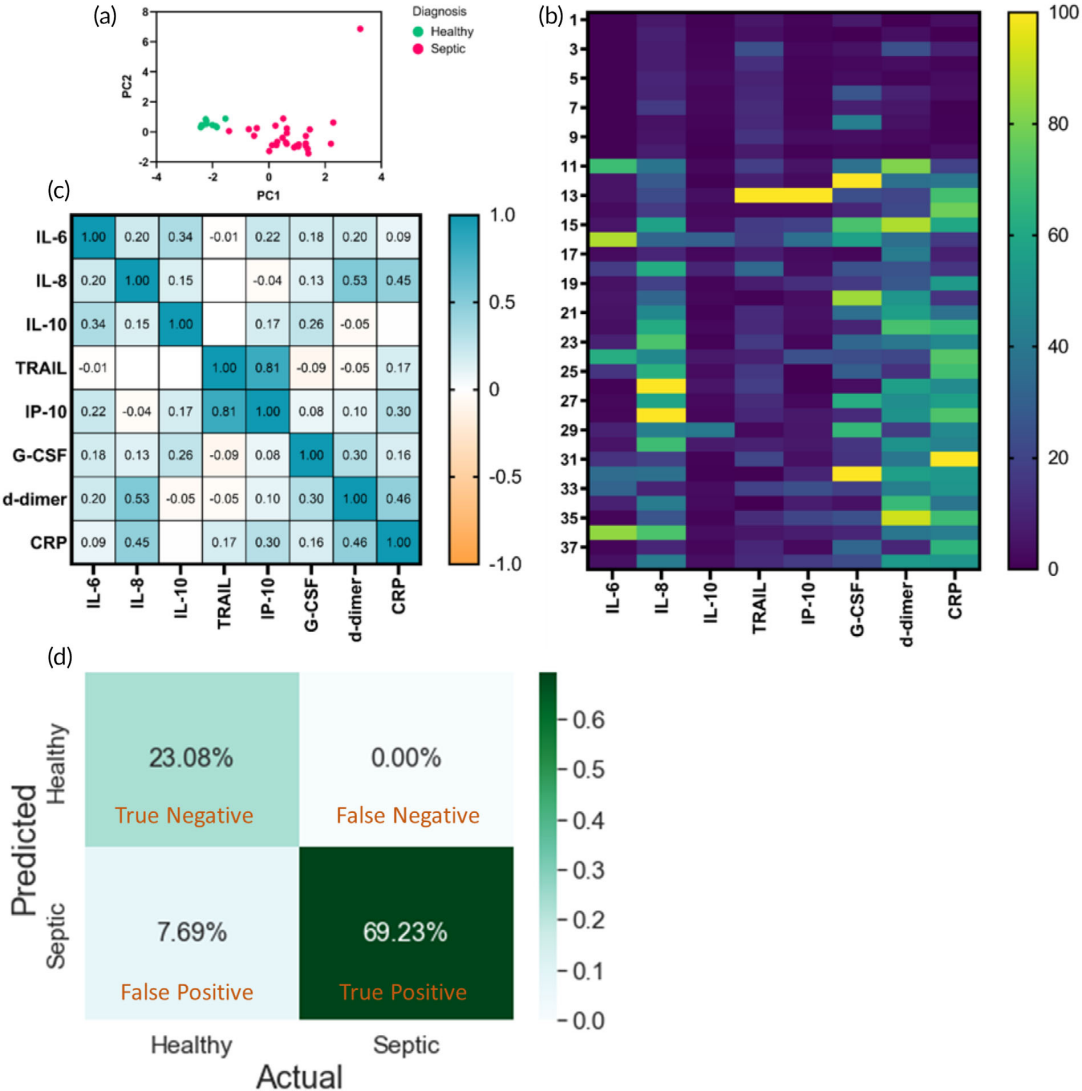
(a) Principal component analysis representing the healthy and septic classification by adding IL‐6, IL‐8, IL‐10, IP‐10, TRAIL, G‐CSF, d‐dimer, and CRP as input variables. (b) Heat map plotted to evaluate the correlation between patient data across multiplexed blood biomarker panel. (c) Correlation analysis between blood panels biomarkers. (d) Confusion matrix showing accuracy of logistic regression machine learning model

Understanding how the effect of combined biomarkers has on the ability to distinguish healthy from the septic patient group, principal component analysis was performed. All eight biomarkers were added as an input to simplify the results and narrow down the results. The summary plot selected three major components PCA1, PCA2, and PCA3 accounting for 29.23%, 22.26%, and 16.99% of the sample variability, respectively. The biomarkers that are responsible to distinguish healthy from septic cohort comprise of PCA1, PCA2, PCA3, PCA4, PCA5, and PCA6 with the cumulative proportion of variance of 94% according to the scree plot (Figure S1). Thus, the scree plot suggests, six components out of the eight are sufficient to provide meaningful insight on the patient status. The loading plot for this data seen in Figure S3 shows that IL‐6, IL‐10, and CRP are positively correlated as they are clustered together. Whereas G‐CSF, IL‐8, and d‐dimer form another cluster and are well correlated with each other. The third cluster formed is between TRAIL and IP‐10 showing a correlation of 0.8. As seen in Figure 7a, for the blood cytokine biomarkers, were able to distinguish healthy control subjects from the septic patient cohort. Heat map analysis shows that the healthy and septic profile is separated with the top one‐fourth section with very low values represented in blue with values toward the lower end of the colored scale bar. All the patients with sepsis showed very high CRP levels followed by d‐dimer, G‐CSF, and IL‐8. Heat map correlation between patients was analyzed to visualize patterns within the different biomarkers as seen in Figure 7b. Data suggest that patients with high IP‐10 levels also had high TRAIL blood concentrations. Next, we evaluated the degree to which certain pairs of biomarkers correlated using Pearson's correlation matrix, seen in Figure 7c. TRAIL and IP‐10 showed the highest correlation of 0.81 followed by d‐dimer and IL‐8 with a coefficient of 0.5. D‐dimer levels have been known to be associated with the activation of pro‐inflammatory cytokine cascade. The paucity of correlation of d‐dimer with anti‐inflammatory cytokine IL‐10 implies that the existence of d‐dimer may represent an imbalance between pro‐inflammatory and anti‐inflammatory cytokines.^25^

On acquiring results from the multiplexed cytokines panel from healthy and septic patients using DeTecT sepsis 2.0 device, we implemented the use of a machine learning model to predict the outcomes. The confusion matrix represented in Figure 7d represents the actual and predicted state using a logistic regression algorithm. The machine learning algorithm used here is the supervised version. We have used a basic logistic regression algorithm to stratify the patient from a healthy and septic state. The logistic regression is a common classification algorithm for two‐class stratification. Since the objective here is to provide decision support for clinicians in determining the probability of a patient being healthy or septic it provides valuable input. We observed the accuracy of logistic regression to be 0.923% sufficient to be confident about the implementation of the algorithm while allowing room for generalization. The true positives here are of 69.23% and true negatives are of about 23.08%. Total false positive which is the type I error is found to be 7.69% and no false‐negative are observed on the test dataset. This builds the confidence on the algorithm that no positive case would be unnoticed. Furthermore, Figure S4 shows other metrics about the classification algorithm. In case of class imbalance, it would be more robust. The f‐1 score gives the overall weighted score for the algorithm and in this case, it is the same as accuracy 0.92. The precision for the healthy group was found to be 0.75 and 1.00 for septic. The individual f‐1 score for the healthy group is 0.86 and 0.95 for the septic one. This shows algorithm can classify the data correctly.

## 3 DISCUSSION

Sepsis is a complicated disease to tackle. With such complicated pathophysiology, differing from individuals based on their immune response, it can be burdensome to track the disease progression. From previous research on the vast availability of biomarkers, certain biomarkers provide a good understanding of how the patients' immune response is changing. Unfortunately relying on a single biomarker for such a complex disease can be of very little use. Therefore, in this study, we combined key biomarkers that provide a snapshot of a patient's immune response. Timing of cytokine release is attributed to disease severity. Proinflammatory immune biomarkers such as IL‐6, IL‐8, and IL‐10 highlight the patient status at the beginning of sepsis. The importance of proinflammatory and anti‐inflammatory cytokine biomarkers has been developed and used for sepsis diagnosis as the paradigm of sepsis pathogenesis has evolved through time with many medical treatments. In patients with severe sepsis, cytokine profiling could be a useful strategy for recognizing various immune response patterns, expressing the diversity of patient groups with identical biological deregulation. These works reflect a multiplexed cytokine analysis in whole blood samples using DeTecT 2.0 device to be able to identify patients with sepsis and the utility of biomarker associations with disease severity. The results in our work confirm that IL‐6, IL‐8, d‐dimer, and CRP are primarily the best indicators of identifying patients with sepsis.

The vast heterogeneity in the immune response of septic patients has made the development of efficient immunotherapies and prediction of infection outcomes that leads to organ failure or death may be the major cause of lack of progress. Therefore, the detection of multiplexed biomarkers has the potential to improve diagnostic efficacy. Furthermore, one of the biggest technological gaps is the availability of rapid detection of these biomarkers at the patient bedside. When a patient suspected of sepsis arrives in the hospital, time is of essence to treat the patient with the right approach. A study reveals early goal‐oriented therapy reduced in‐hospital mortality as compared to conventional care (30%–46%).^26^ Additionally, in septic shock, starting antibiotics within 1 h improves survival, whereas every hour antibiotics are delayed reduces survival by 8%. Choosing the right set of biomarkers may provide valuable insight into understanding the pathophysiology of sepsis in each patient. Cytokines are known to be the first biomarkers to respond to inflammation.^27^ This is also reflected by the results where IL‐6 and IL‐8 have shown a significant difference between healthy and septic patient cohorts, seen in Figure 5.

It is observed that 20%–25% of patients with sepsis have disseminated intravascular coagulation (DIC) which may lead to organ failure, thereby significantly increasing the risk of mortality.^28^ The activation of a coagulation deluge is a typical and early occurrence in septic patients with infection, and several molecules involved in this process are also key inflammatory response amplifies. Fibrinolysis is activated by d‐dimer which causes coagulation. The role of d‐dimer is significant in identifying the severity of immune response, as d‐dimer signifies the level of blood coagulation which in turn can reflect severity in host immune response, leading to organ failure.^29^ This work showed results in line with the results shown with the previous study of d‐dimer in sepsis. A higher mortality rate and hospitalization were found to be correlated with patients with higher d‐dimer levels8. The level of d‐dimer in our septic patient cohort was nearly 10 times higher than of healthy patient group^9^, ^10^ and correlated moderately with CRP, thus becoming a potential biomarker for identifying infected individuals with a high risk of mortality in a simple and quick manner. The levels of CRP have known to be markers of sepsis. CRP levels increase within the first 6 h of the infection. Although it is a generic biomarker for inflammation, the incidence of sepsis is much higher.^30^ CRP is also used as a standard for measuring sepsis as part of the clinical standard, or blood culture. CRP is also a marker used to test for adverse outcomes with COVID‐19.^11^ The concentration difference between healthy and septic patient cohorts is almost 200 times, which indicates a characteristic response to the infection. Several studies have shown that increased CRP concentration especially in patients in ICU has an increased risk of organ failure and higher chances of mortality.^12^, ^13^ IL‐6 is an essential cytokine that is a key activator for acute phase response, particularly, triggering the production of CRP, a well‐known pro‐inflammatory marker for atherothrombotic vascular disease14. Tracking IL‐6 in connection with CRP has been shown to have prognostic value for early detection of sepsis.^31^ The threshold value of IL‐6 and CRP was 1.6 pg/ml and 2619 ng/ml which was in line with previous studies.^32^, ^33^ IL‐6 is also known to be one of the first mediators to provocate the cytokine storm in patients with sepsis and COVID‐19.^34^ Therefore, monitoring the levels of IL‐6 along with other pro‐inflammatory markers provides evidence of how the patient's immune response is reacting during the infection. Diagnostic accuracy of a single marker in a complex disease like sepsis is difficult to predict the dynamic state of the patient.^35^ The novel combination of biomarkers showcasing various immune response phases within the patient provides valuable information to the clinicians to make crucial decisions on therapeutics. For instance, if the presenting patient shows higher proinflammatory levels of biomarkers, the clinician can provide immunosuppressants to dial down the immune response, and avoid the catastrophic event of a cytokine storm, which could lead to uncontrollable side effects like multiple organ failure, or even death. Herein IL‐10 plays a crucial role to determine the state of the immune response. IL‐10 is known to act as a double‐edged sword during infection as it acts based on the body's feedback. For instance, If the patient is in the state of a hyperimmune state, IL‐10 is triggered as an anti‐inflammatory signal to lower the over‐responsive effects of the inflammatory response. Whereas, if the body is in the state of immunosuppression, IL‐10 triggers the necessary biomarkers to activate the pro‐inflammatory response, to curb the infection in the body during infection. The right role of IL‐10 is unknown during sepsis, but as it has a dual nature, the diagnostic accuracy can be affected. Therefore, it is necessary to club IL‐10 along with other pro‐inflammatory markers to completely understand the nature of the individual biomarker. Overproduction of IL‐10 has been associated with severe outcomes and mortality.^15^ IL‐10 suppresses the activity of proinflammatory cytokines and chemokines including IL‐6, IL‐8, IP‐10, and G‐CSF.^16^ Its major role is to limit damage to the host. But in doing so, it also grants a free pass to the pathogen to multiple or sustains within the host, thereby harming the host eventually. Therefore, if IL‐10 is expressed at an inopportune time, such as too early during virulent infection, or too late during avirulent infection, it can cause overwhelming infection or severe tissue damage.^17^ This work is in line with the research done previously where 75% IL‐10 production is at 34 pg/ml up to 272 pg/ml in septic patient cohort.^18^ Progression of TRAIL and IP‐10 has been researched over different patient groups and has been said to be distinguishing factors for bacterial and viral sepsis. Especially, lower levels of TRAIL show more severe outcomes.

Detection of biomarkers in whole blood is a completely different arena compared to regular standard diagnostics. There are a few advantages of exploring blood as a biofluid for ease in detection. This work focuses on the potential of using a point‐of‐care device capable of measuring eight biomarkers simultaneously in a small sample of whole blood within 5 min.

In a clinically relevant environment, effective detection and prediction of the patient at risk of developing sepsis are key for successful management. Integrating the machine learning model with the multiplexed cytokine results in blood enables a unique way to predict the possibility of a patient with sepsis with high accuracy (AUC >0.92). The application of machine learning‐based predictive technology might aid medical decision‐making by adding new components to assist proper and early diagnosis of patients with sepsis.

Even though a combination of cytokines shows high accuracy for predicting patients with sepsis, our work has a few limitations. The limited sample size restricts the extent to which our findings can be extended to other patient cohorts. Furthermore, only a single time point cytokine measurement was considered, and although blood measurement is crucial for early prognosis, it may not elucidate the depth of the influence of cytokines in disease etiology as compared to those offered by sequential measurements. However, specific cytokine accuracy was high for predicting the patient state and disease severity. Although it is tempting to presume that variations in cytokine concentrations are linked to the pathophysiology of organ failure, we believe, no single cytokine can be credited for the entire severity of the disease. Cytokine concentrations in blood could also be elevated merely as indicators of tissue injury, without necessarily playing a direct role.

The multiplexing capability of DeTect 2.0 device directly in whole blood at the patient bedside provides opportunities to create a panel of sepsis biomarkers that includes well‐studied cytokine biomarkers with good prognostic value along with other biomarkers to accommodate for similar diseases like COVID‐19 to expand the potential application of the device. To gain a comprehensive understanding of complex interactions that occur during disease progression, we acknowledge that newer techniques can benefit, rather than studying the individual effect of the chosen biomarkers.

Apart from the current limitations, DeTect Sepsis device 2.0 has several advantages over existing analytical techniques. Firstly, the high detection sensitivity of DeTecT sepsis 2.0 device coupled with a wide dynamic range and good specificity provides rapid and accurate results to improve sepsis stratification. Next, the miniaturized portable device can be used anywhere, from the patient bedside to the emergency department or even in an ambulance. Secondly, a low sample requirement equivalent of two drops of blood (~100 μl) provides rapid information on a panel of eight useful biomarkers which can provide valuable insights on the patient's host immune response at that instant. Lastly, integrating a machine learning algorithm with the multiplexed cytokine panel offers the user (e.g., clinicians) valuable insights and can be a valuable asset as a clinical decision support system for improved patient outcomes.

To date, there is no point‐of‐care device that can rapidly assess the biomarker concentrations without sample dilution in whole blood with low sample volume (~100 μl). This translational research work would support and benefit the clinical community in rapidly assessing the state of the patient's immune response at the time of intervention, thus providing real‐time valuable information. In conclusion, simultaneous detection of eight cytokine biomarkers combined with a machine learning model can reveal complicated cytokine patterns reflecting systemic response linked to severe sepsis, organ failure, and death. Based on the cytokine profiles provided by the DeTecT sepsis 2.0 device, clinicians can assess disease severity and forecast distinct clinical presentations and outcomes.

## 4 EXPERIMENTAL SECTION

### 4.1 Experimental design

This work is designed to validate the performance of the developed DeTecT sepsis 2.0 device for simultaneous detection of key sepsis biomarkers directly in whole blood. To validate the efficacy of the sensor against whole blood samples, 30 whole blood patient samples were procured from Discovery life science (Atlanta), which was declared positive for sepsis using reference laboratory standard technique. Parallelly, 10 healthy whole blood samples were procured from Carter blood bank (Texas) as positive control samples. The sample size calculation is shown in Supporting Information. Dithiobis succinimidyl propionate (DSP) along with Dimethyl sulfoxide (DMSO) solvent, phosphate Buffer Saline (PBS), SuperBlock was procured from Thermo Fisher Scientific (USA). Multiplexed biomarkers used for this work was Interleukin‐6 (IL‐6), Interleukin 8 (IL‐8), Interleukin‐10 (IL‐10), interferon‐gamma induced protein‐10 (IP‐10), TNF‐related apoptosis‐inducing ligand (TRAIL), Granulocyte‐colony stimulating factor (G‐CSF), d‐dimer, C‐reactive protein (CRP). All the antigens and antibodies were purchased from Abcam. All the stock proteins were aliquoted and stored in −20°C until further use. To prevent denaturing the proteins, none of the proteins underwent more than three freeze–thaw cycles. Antibodies were diluted in PBS to get them to their optimized concentrations before use.

### 4.2 DETecT 2.0 sensor with EnLiSense's rapid electro analytical device platform

The DeTecT sepsis 2.0 device consists of a uniquely designed sensor with 16 independent electrodes on a single PCB platform. The gold deposited electrodes are equally spaced on the PCB surface to provide uniform fluid flow. All the 16 working electrodes is deposited with a thin layer of zinc oxide (ZnO) uniformly to attain high sensitivity. The deposited sensor is then mounted on a handheld reader device which encompasses the portable electronics with EIS module for the signal detection. The reader is compatible to support both wired and wireless communication. The detection mechanism is based on the EIS framework where subtle changes that occur at the electrode solution interface can be detected by applying a small input voltage over a frequency spectrum. The sensor requires a sample of approximately 100 μl which is equivalent to two drops of blood. The detection occurs immediately, and results can be read within 5 min. Shorter measurement times ensure the blood does not clot and measurement is taken promptly. Assay development, sensor characterization, and validation were performed using similar protocols followed in our previous work.^24^ To scale the capability of the sensing device to measure eight biomarkers, we had to activate eight channels as opposed to five channels that were used in our previous work. The upgraded sensing platform had to be calibrated to measure the new biomolecules simultaneously. While upgrading the biomarker panel from 5–8 biomarkers was not an issue as the sensing platform was robust, the assay had to be optimized to account for whole blood analysis. While plasma behaves almost Newtonian, whole blood has distinct non‐Newtonian features, which are mostly explained by erythrocyte clumping at low shear rates, deformability, and a propensity to align with the flow field at high shear rates. To minimize the effect of noise while measuring the given sample, we focused on a smaller frequency range (80 –1000 Hz) while using electrochemical impedance spectroscopy. Narrowing the frequency range in the lower frequency regime helped extracting the signal response which captured the biomolecular interaction occurring at the sensing interface. Assay optimization included lowering the detection time to avoid sample deterioration and signal interference due to blood clotting on the sensing platform. Hence, the assay optimization was critical to ensure rapid detection and not allow the blood to clot on the sensor and provide erroneous results. Machine learning algorithms were implemented to visualize and predict the healthy and septic patient groups using supervised and unsupervised algorithms. Additional information on device comparison between previous version can be found in Supporting Information.

### 4.3 Statistical analysis

The Graphpad software was used to perform statistical analysis (GraphPad Software Inc., La Jolla, CA). *p 0.05, **p 0.01, ***p 0.001, ****p 0.0001, ns: nonsignificant, *p 0.05, **p 0.01, ***p 0.001, ****p 0.0001. Unless otherwise noted, data is provided as mean *SEM* for n = 3 replicates. For comparisons between three or more groups, a one‐way analysis of variance (ANOVA) was performed. In the cross‐reactive investigation, the *T*‐test was utilized to examine the importance of signals versus serum albumin. Nonparametric unpaired Mann–Whitney tests were used to compare the healthy and septic cohorts. Biomarkers were compared using two methodologies, and the difference between them was determined using Bland–Altman analysis. Wilson/Brown method using GraphPad Prism was used to assess DETecT sepsis 2.0 device specificity and sensitivity to identify healthy from the septic patient group using receiver operating parameters. Along with many additional input criteria, principal component analysis (PCA) was utilized to visually evaluate if a healthy cohort could be distinguished from septic patients. Additional information on the DETecT Sepsis device with five biomarkers in plasma has been described in Figure S4. Logistics regression is the basic algorithm used for classification. Logistics regression is the take on linear regression with a sigmoid function. Before applying the logistics, regression data cleaning has been done. The data cleaning part of the pipeline would take care of any missing values or bad values that may affect the prediction. After the basic data cleaning and exploration, the next task was to build a machine learning model. We have done it with two different phases supervised and unsupervised. The supervised version of the model was used to test the prediction. The scikit learn‐based logistics algorithm was used for the implementation of the clean data. The results obtained are explained in the results section. For the unsupervised version, the main objective was to find the natural groupings within the dataset. For the unsupervised version, the first dimensionality reduction has been performed. The components obtained from the analysis are used as the input parameter for the unsupervised clustering algorithm. The entire dataset of 40 was divided into a 70:30 ratio. 70% is used for training and the remaining 30% has been used for the test. The confusion matrix is calculated based on 30% of the dataset. Given the nature of the restricted dataset, we have used the basic version of the logistic regression package made available from scikit. The PCA output has been plotted with the color by the groupings which are previously known. The clustering algorithms have been implemented using the scikit learn package. Given the nature of the dataset, we use the basic clustering algorithm such as k‐means, agglomerative and BIRCH algorithm. The scoring criteria used here is the silhouette scoring method to understand the inter and intra distance between the clusters.

## CONFLICT OF INTEREST

Drs. Shalini Prasad and Sriram Muthukumar have a significant interest in EnLiSense LLC, a company that may have a commercial interest in the results of this research and technology. The potential individual conflict of interest has been reviewed and managed by The University of Texas at Dallas, and played no role in the study design; in the collection, analysis, and interpretation of data; in the writing of the report, or in the decision to submit the report for publication.

## AUTHOR CONTRIBUTIONS

**Ambalika S. Tanak:** Conceptualization (equal); data curation (lead); formal analysis (lead); methodology (equal); validation (lead); writing – original draft (lead); writing – review and editing (lead). **Abha Sardesai:** Formal analysis (supporting); methodology (supporting); writing – review and editing (supporting). **Sriram Muthukumar:** Conceptualization (equal); data curation (supporting); formal analysis (supporting); resources (equal); supervision (equal); writing – review and editing (supporting). **Shalini Prasad:** Conceptualization (equal); methodology (equal); resources (lead); supervision (equal); writing – review and editing (supporting).

## Supporting information


**Appendix** S1: Supporting Information

## Data Availability

Research data are not shared.
