# Supplementary material for: Simultaneous detection of sepsis host response biomarkers in whole blood using electrochemical biosensor
Source: Bioeng Transl Med. 2022 Mar 17;7(3):e10310. doi: 10.1002/btm2.10310 (PMC9471994; doi:10.1002/btm2.10310)
Supplement: Supplementary file 1 — Appendix S1: Supporting Information [file BTM2-7-e10310-s001.docx]

**Simultaneous Detection of Sepsis Host Response Biomarkers in Whole Blood Using Electrochemical Biosensor**

Ambalika S Tanak^1^, Abha Sardesai^2^, Sriram Muthukumar^3^*, and Shalini Prasad^1^*

^1^Department of Bioengineering, University of Texas at Dallas, TX.

^2^Department of Computer engineering, University of Texas at Dallas, TX.

^3^EnLiSense LLC, 1813 Audubon Pond Way, Allen, TX, 75013.

***Corresponding Author(s): Shalini Prasad**

Address: 800 W. Campbell Rd. BSB 11,

Richardson, TX, USA 75080

Email: [shalini.prasad@utdallas.edu](mailto:shalini.prasad@utdallas.edu)

Phone: 972-883-4247

Sriram Muthukumar

Address: 1813 Audubon Pond Way,

Allen, TX, USA 75013

Email: [sriramm@enlisense.com](mailto:sriramm@enlisense.com)

**Supplementary information**

Table S1 Individual mean bias values

| **Biomolecule** | **Bias**  (pg/mL) | **SD of Bias**  (pg/mL) | 95% Limits of Agreement  (pg/mL) |
| --- | --- | --- | --- |
| IL-6 | \| 14.42 \| \| --- \| | \| 49.35 \| \| --- \| | -82.31 to 111.1 |
| IL-8 | 75.59 | 279 | -471 to 622.4 |
| IL-10 | 0.003 | 2.19 | -4.2 to 4.3 |
| IP-10 | -1.86 | 22.44 | -45.85 to 42.11 |
| TRAIL | 0.88 | 3.78 | -6.5 to 8.3 |
| G-CSF | 0.74 | 5.57 | -10.18 to 11.66 |
| D-Dimer (ng/mL) | -16.63 | 113 | -238.2 to 204.9 |
| CRP (ng/mL) | 410 | 3835 | -7107 to 7927 |


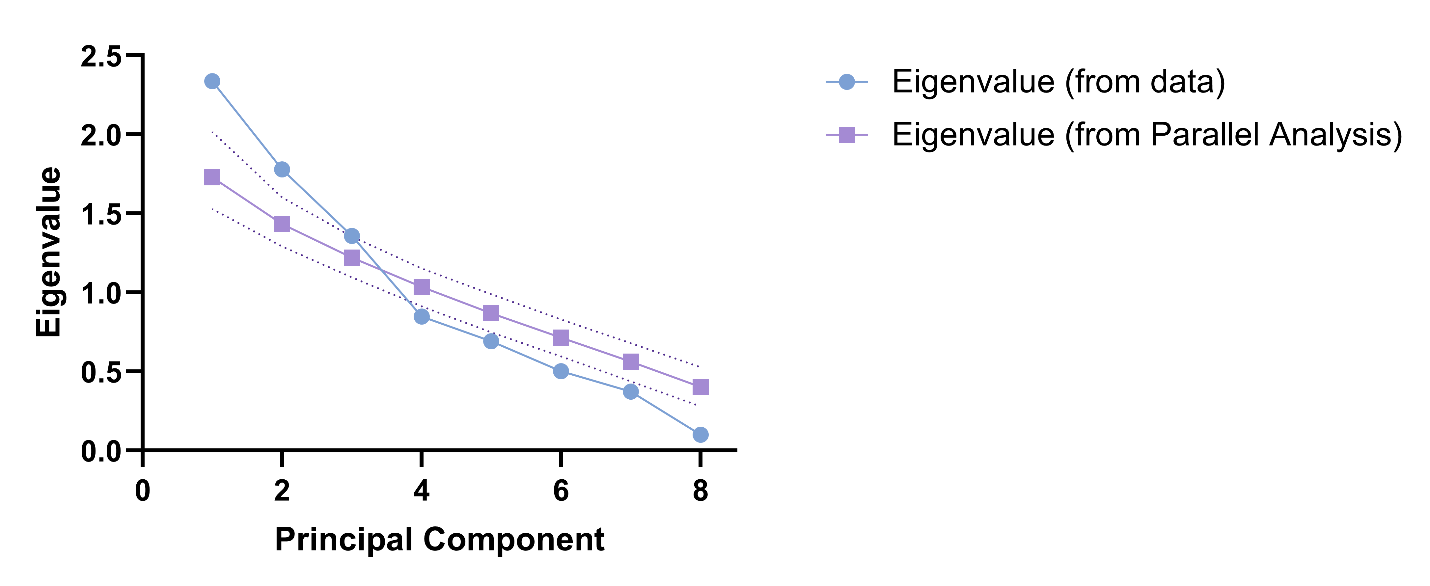


Figure S1 scree plot


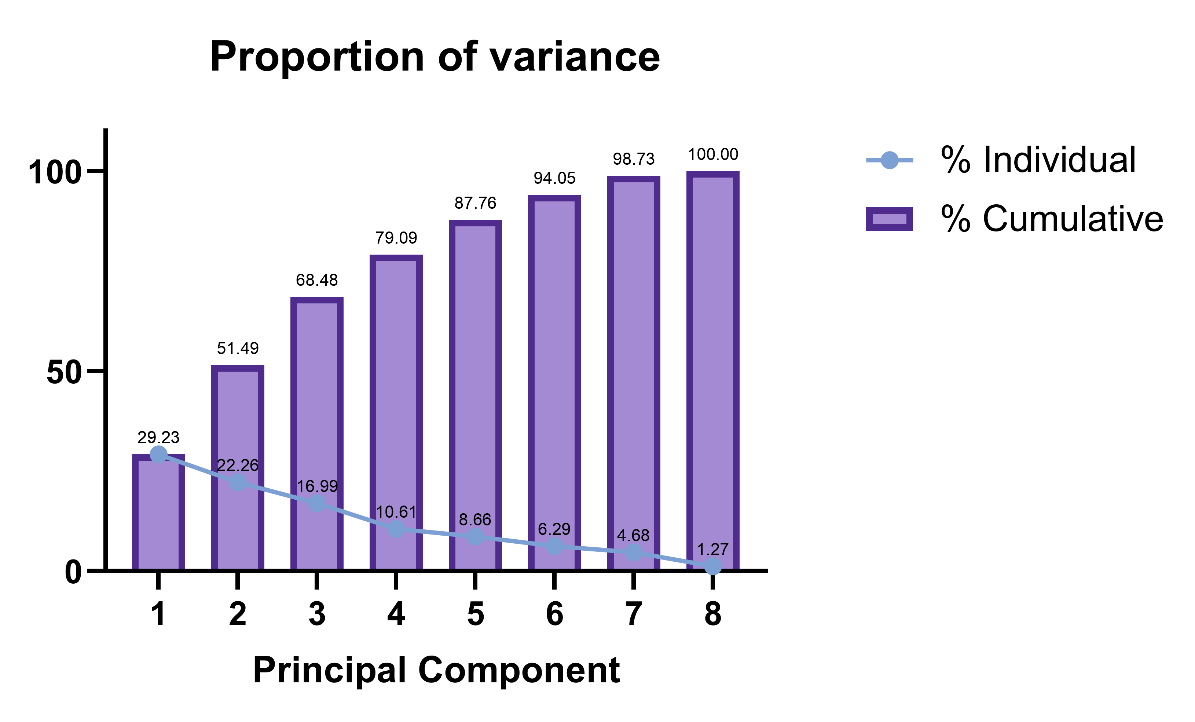


Figure S2 Proportion of variance for whole Blood


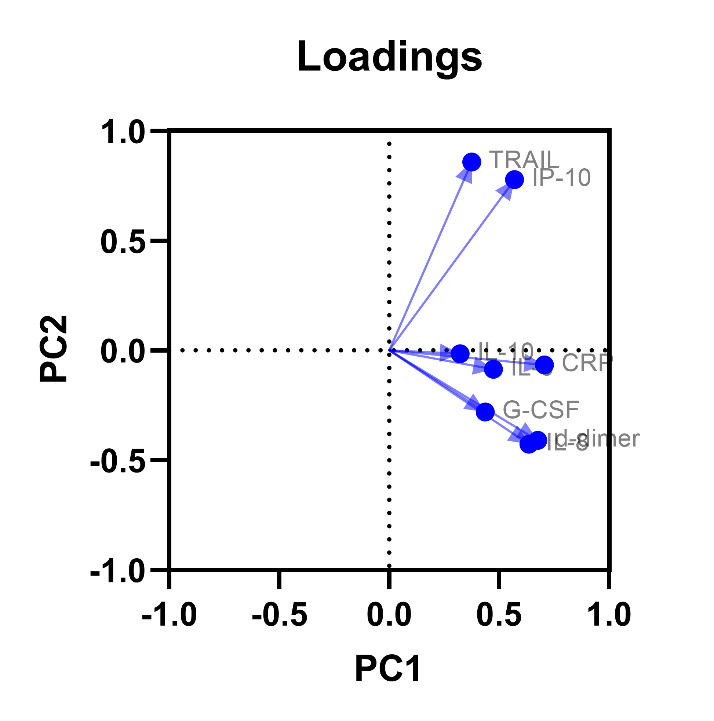


Figure S3: Loading plot for IL-6, IL-8, IL-10, IP-10, TRAIL, G-CSF, d-dimer, and CRP.


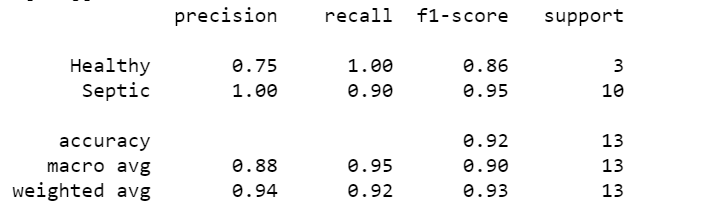


Figure S4 classification algorithm





Figure S4 PCA plot for the previous version of DETecT Sepsis device that measures five biomarkers in plasma.

The PCA plot with five biomarkers is shown in figure S4 using the previous version of the DETecT Sepsis device that compares the performance of five biomarkers measured in plasma with the upgraded panel with the addition of eight biomarkers measured in whole blood. There is an overlap between the healthy and septic patient samples seen, whereas in comparison to the eight-biomarker panel PCA shows definite distinguished boundary between the healthy and septic patients’ population, indicating that the additional biomarkers aid in patient classification.

The confusion matrix featuring five biomarkers in our previous publication^1^ has a true positive value of 60.71% and a true negative value of 36.71%. The false positive rate or Type 1 error as well as type 2 error (false negative) is approximately 1.69 %. Whereas the performance of the sensing device improves with the addition of three biomarkers to the panel by eliminating the probability of type 2 error. It also increases the true positive value to 69.23% as seen in figure 7D and has a true negative value of 23.08%. This means, when the device uses eight biomarkers, it builds the confidence on the algorithm that no positive case would be unnoticed. The presence of type II error with five biomarkers might be an issue while identifying the actual positive cases hence having three extra biomarkers would be helpful.

**Sample size calculation**

The power calculations based on the pilot study with a power of 0.8, alpha (probability of type 1 error) of 0.05 and beta (probability of type 2 error) of 0.2, was determined to be 10 samples each for healthy and septic cohorts. Keeping this as reference, we used 10 healthy samples and 30 septic patient samples for our study to make a total sample set of 40 for the whole blood analysis. The power calculation for sample size determination is given below.


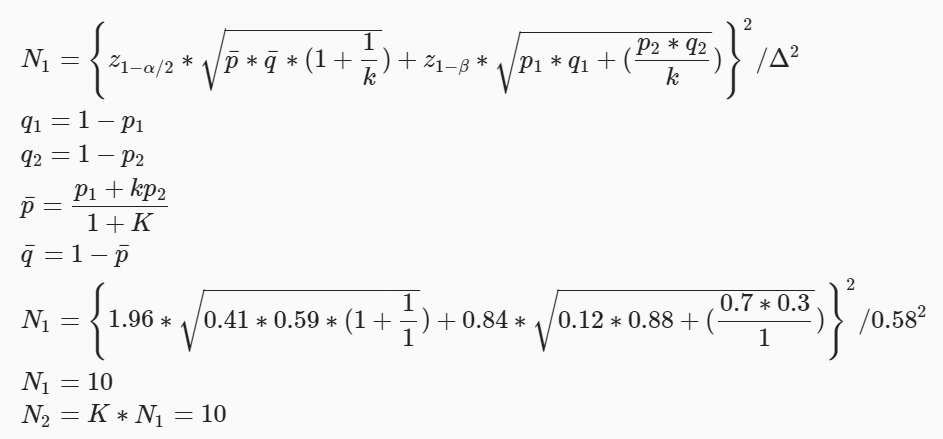


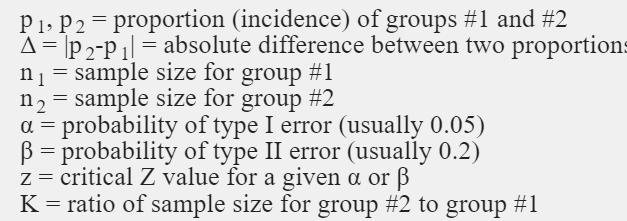


**References**

1. Sardesai AU, Tanak AS, Krishnan S, et al. An approach to rapidly assess sepsis through multi-biomarker host response using machine learning algorithm. *Scientific Reports 2021 11:1*. 2021;11(1):1-10. doi:10.1038/s41598-021-96081-5
